# Nutrition Strategies for Next‐Generation Incretin Therapies: A Systematic Scoping Review of the Current Evidence

**DOI:** 10.1111/obr.70079

**Published:** 2026-01-07

**Authors:** Marie Spreckley, Cara F. Ruggiero, Adrian Brown

**Affiliations:** ^1^ MRC Epidemiology Unit University of Cambridge Cambridge UK; ^2^ Centre for Obesity Research University College London Longdon UK; ^3^ Bariatric Centre for Weight Management and Metabolic Surgery University College London Hospital NHS Foundation Trust London UK; ^4^ National Institute for Health and Care Research University College London Hospitals Biomedical Research Centre London UK

**Keywords:** GLP‐1 receptor agonists, nutrition, semaglutide, tirzepatide

## Abstract

Next‐generation incretin therapies, including semaglutide and tirzepatide, have transformed obesity and Type 2 diabetes management. However, evidence‐based nutritional strategies to support safe and effective use of these agents remain limited. To address this gap, we conducted a systematic scoping review across five databases of studies published between January 2015 and April 2025 to map and appraise clinical trials incorporating nutritional interventions or dietary assessments during semaglutide or tirzepatide therapy in adults with obesity or Type 2 diabetes. Eligible studies included adults receiving semaglutide or tirzepatide with either an active dietary intervention or measured nutrition‐related outcomes. Study quality was assessed using established tools. Twelve studies were included: 10 randomized controlled trials, one non‐randomized comparative study, and one cross‐sectional observational study. Interventions ranged from structured very‐low‐energy or ketogenic diets to general lifestyle counseling and observational dietary assessments. Across studies, energy intake decreased by 24% to 39%, but lean tissue loss accounted for up to 40% of total weight reduction. Only three studies involved nutrition professionals, and systematic assessment of protein or micronutrient intake was rare. One observational study found widespread nutrient inadequacies and limited access to dietetic support. Despite the effectiveness of semaglutide and tirzepatide for weight loss, evidence on optimal dietary strategies is sparse. Early dietitian involvement, high‐protein, nutrient‐dense diets, and routine nutritional monitoring should be prioritized. Robust trials are needed to define best practice for integrating dietary care alongside pharmacotherapy.

**Abbreviations**
AOM: anti‐obesity medication
BMI: body mass index
DASH: Dietary Approaches to Stop Hypertension
DRI: dietary reference intake
GI: gastrointestinal
GIP: glucose‐dependent insulinotropic polypeptide
GLP‐1 RA: glucagon‐like peptide‐1 receptor agonist
HEI: Healthy Eating Index
LCD: low‐calorie diet
LEKT: low‐energy ketogenic therapy
MD: Mediterranean diet
PICOS: Population, Intervention, Comparator, Outcomes, Study design
RCT: randomized controlled trial
RDN: registered dietitian nutritionist
RoB 2: Cochrane risk‐of‐bias tool for randomized trials
ROBINS‐I: Risk of Bias in Non‐randomized Studies—of Interventions
T2D: Type 2 diabetes
VLCD: very‐low‐calorie diet

## 1 Introduction

The introduction of next‐generation incretin therapies, including glucagon‐like peptide‐1 receptor agonists (GLP‐1 RAs) and dual glucose‐dependent insulinotropic polypeptide/GIP/GLP‐1 RAs, marks a significant advance in the management of obesity and Type 2 diabetes mellitus (T2D). Semaglutide and tirzepatide have demonstrated weight‐loss efficacy comparable to bariatric surgery, with mean reductions of up to 20.9% in body weight, along with other metabolic benefits, across pivotal trials [1, 2, 3, 4]. By mimicking endogenous incretin hormones, these agents suppress appetite, increase satiety, and reduce food cravings, resulting in substantial reductions in energy intake [5, 6, 7]. However, their effects on overall diet quality and nutrient intake remain poorly understood [7].

As semaglutide and tirzepatide become increasingly integrated into routine clinical obesity care, clinicians face new questions regarding their use within multidisciplinary treatment approaches. These include the potential nutritional consequences of marked appetite suppression and the risk of nutrient inadequacy. Although these agents are now widely prescribed, there remains limited practical guidance or statements on dietary support during treatment [8, 9, 10, 11, 12]. Gastrointestinal side effects, such as nausea, early satiety, and altered taste, are common and may compromise dietary intake, increasing the risk of deficiencies [12].

Available data indicate reductions in caloric intake of 16%–39% during incretin therapies, yet few studies have examined diet quality, protein intake, or micronutrient adequacy [5, 6, 13, 14]. Very‐low‐ and low‐energy intakes (≤ 800 and 1200 kcal/day, respectively), when implemented without clinical supervision or in individuals with pre‐existing nutritional vulnerabilities, may increase the risk of inadequate protein intake, dietary quality, and micronutrient deficiencies [11, 15]. This is particularly concerning given that lean body mass can constitute up to 40% of total weight lost during treatment [11, 16]. In the context of sarcopenic obesity, nutritional strategies must prioritize adequate protein, micronutrient, fiber, and fluid intake to preserve lean mass and support metabolic health [17].

To date, most dietary advice during incretin therapy has focused on calorie reduction, with little emphasis on nutritional adequacy [1, 9], though recent published guidelines have started to highlight this importance [10]. Moreover, the design and delivery of dietary components in clinical trials have been highly variable, with inconsistent macronutrient goals and limited professional oversight [18, 19].

This systematic scoping review aimed to synthesize current evidence on nutrition components reported in clinical trials focusing on semaglutide and tirzepatide in adults with obesity or T2D as well as prospective and observational studies. Particular focus was placed on the types and delivery of nutrition support, including structured interventions, lifestyle counseling, or observational assessments, and evaluates their effects on nutritional outcomes, including energy intake, body composition, protein adequacy, and patient experiences. It also examines whether dietitian involvement influences adherence and outcomes and highlights key evidence gaps to inform future trials and clinical guidelines.

## 2 Methods

### 2.1 Search Criteria

A systematic literature search was conducted on 26 April 2025 across five databases (PubMed, Embase, Scopus, Web of Science, and Google Scholar) to identify peer‐reviewed studies published between January 2015 and April 18, 2025. The search focused on adult populations (≥ 18 years) using next‐generation incretin therapies (specifically semaglutide or tirzepatide) that either included an active nutritional intervention or assessed nutritional intake. Search terms included combinations of “GLP‐1 receptor agonist,” “semaglutide,” “tirzepatide,” “diet,” “nutrition,” and “counselling,” with Boolean operators and field‐specific filters (see Supporting Information). Only human studies published in English were included.

Liraglutide was excluded as this is a once‐daily injection and, due to its slower and more modest effects on weight loss compared with semaglutide and tirzepatide, was outside the scope of this review.

A total of 63 records were retrieved. After removing duplicates, 43 unique articles were screened independently by two authors (M.S. and C.F.R.). Studies were excluded if they involved animal or preclinical models (*n* = 12), were not primary research (e.g., reviews or commentaries) (*n* = 9), addressed only modeling or cost‐effectiveness (*n* = 5), were case reports (*n* = 3), or were protocols or ongoing trials without results (*n* = 2). Disagreements were resolved by discussion between all three authors (M.S., C.F.R., and A.B.).

Twelve full‐text articles were included: 10 randomized controlled trials (RCTs), one prospective non‐randomized comparative study, and one cross‐sectional observational study. All met the predefined eligibility criteria: adult participants treated with semaglutide or tirzepatide, an active nutritional component or structured dietary assessment, and outcomes related to nutrition (such as energy intake, body composition, nutrient adequacy, or patient‐reported dietary experience).

**FIGURE 1:**
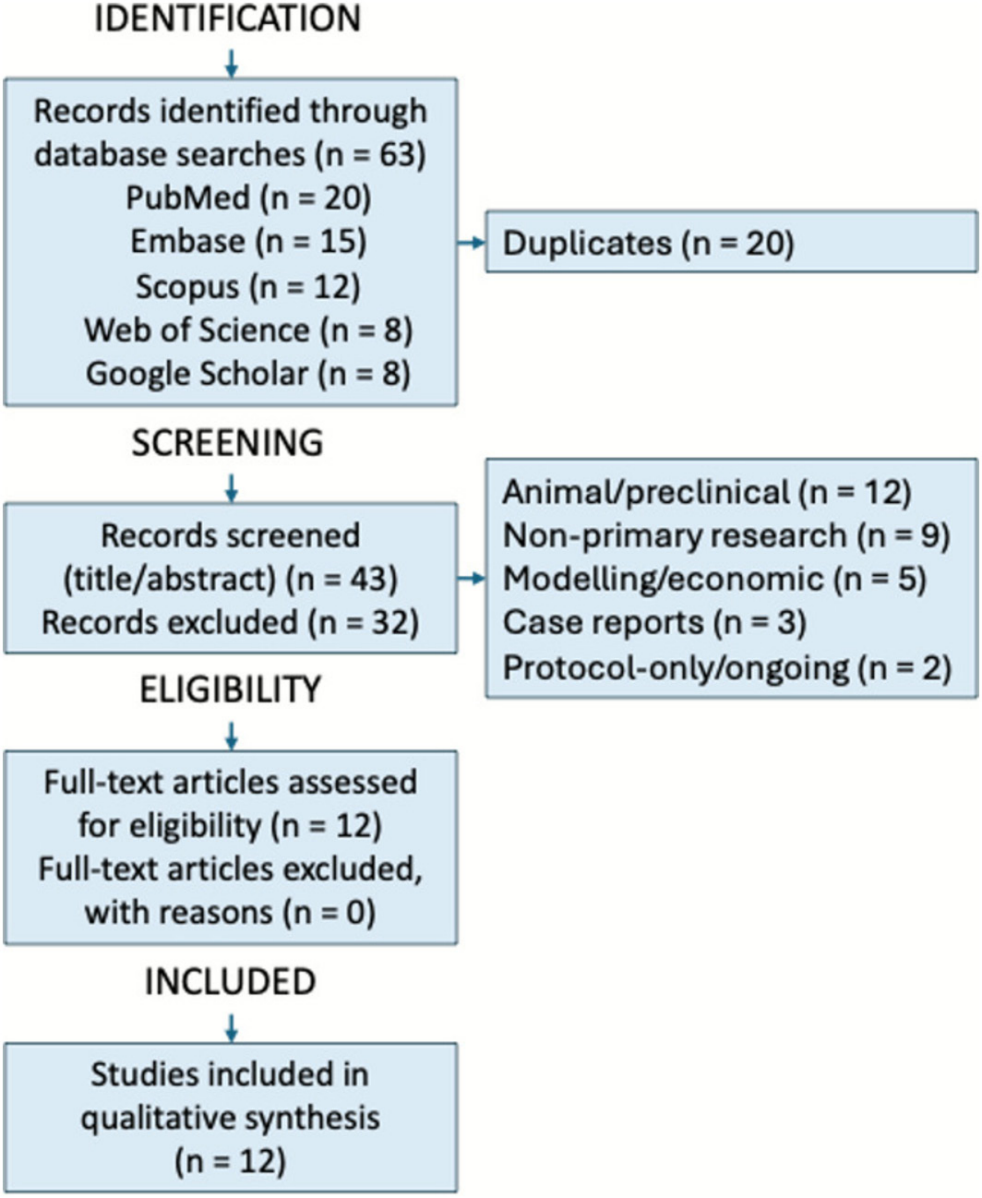
PRISMA flow diagram of study selection. Description: Flow diagram of study selection (records identified = 63; studies included = 12).

The selection process is illustrated in Figure 1. Study characteristics are summarized in Table S1 using the PICOS framework (see Supporting Information).

### 2.2 Study Selection

Data extraction was conducted independently by the first two authors (M.S. and C.F.R.) using a standardized template. Extracted data included study design, participant characteristics, GLP‐1 RA regimen, nutrition intervention features (e.g., caloric goals, macronutrient targets, counseling structure, and dietitian involvement), intervention duration, and key nutrition‐related outcomes. Where reported, we extracted any assessment of overall diet quality and the instrument used. Only one study collected 3‐day diet records and compared intakes with dietary reference intakes (DRIs) and MyPlate food group targets [20]; no included study applied validated composite diet‐quality indices such as the Healthy Eating Index (HEI) or Mediterranean Diet Score [21, 22].

Studies were grouped into three categories:

1. Structured dietary protocols (e.g., very‐low‐calorie diets [VLCDs], ketogenic diets, or prescribed hypocaloric regimens).
2. Lifestyle counseling without prescriptive dietary plans.
3. Observational assessments of dietary intake, appetite, or nutritional markers.

One non‐randomized comparative study [23] and one cross‐sectional study [20] were included alongside the 10 RCTs. The cross‐sectional study was retained due to its detailed analysis of habitual dietary intake and nutrient adequacy in GLP‐1 RA users, which aligned with the review objectives.

Given the heterogeneity of interventions and outcomes, no meta‐analysis was performed. Findings were narratively synthesized to describe patterns in nutritional support and its reported effects on energy intake, nutrient adequacy, body composition, and patient experience. The inclusion of observational and non‐randomized studies allowed broader insight into nutritional challenges during GLP‐1 RA therapy.

Risk of bias was assessed using the Cochrane risk‐of‐bias tool for randomized trials (RoB 2) [24] and the Risk of Bias in Non‐randomized Studies of Interventions (ROBINS‐I) tool [25]. Two authors independently applied domain‐level judgments, with discrepancies resolved by consensus.

## 3 Results

Twelve full‐text articles met the inclusion criteria. These comprised 10 RCTs, one prospective non‐randomized comparative study [23], and one cross‐sectional observational study [20]. Sample sizes ranged from 15 to 670 participants, with intervention durations ranging from 12 to 68 weeks in the interventional studies. Most studies included adults with obesity or T2D, although two studies also included people with prediabetes [26] or knee osteoarthritis [27].

The risk of bias assessment was conducted by two authors (MS, CFR). Seven of the 10 RCTs were judged to have a low risk of bias based on the RoB 2 tool [5, 6, 13, 14, 26, 28, 29]. Three trials had some concerns due to either small sample size or unblinded counseling [27, 30, 31]. The non‐randomized comparative study was judged to have a high risk of bias [23], and the cross‐sectional study was assessed as having serious risk [20] using an adapted ROBINS‐I framework. Table 1 summarizes the risk‐of‐bias judgments for each study.

**TABLE 1:** Risk‐of‐bias assessment summary.

Study	Random sequence and allocation concealment	Blinding: Participants and personnel	Blinding: Outcome assessment	Incomplete outcome data (attrition)	Selective reporting	Overall RoB
Schiavo et al. 2025	High (non‐randomized)	High (open‐label)	Moderate	Low	Low	High
Wadden et al. 2021, STEP 3	Low	Low	Low	Low‐moderate (~7%–8% attrition)	Low	Low
Mu et al. 2024, STEP 7	Low	Low	Low	Low	Low	Low
McGowan et al. 2024, STEP 10	Low	Low	Low	Low	Low	Low
Anyiam et al. 2024	Low	Moderate (open‐label; small N)	Low	Low	Low	Moderate
Bliddal et al. 2024	Low	Moderate (pragmatic counseling)	Low	Low	Low	Moderate
Aronne et al. 2024, SURMOUNT 4	Low	Moderate (pragmatic counseling)	Low	Low	Low	Moderate
Gibbons et al. 2020	Low	Low	Low	Low	Low	Low
Blundell et al. 2017	Low	Low	Low	Low	Low	Low
Friedrichsen et al. 2021	Low	Low	Low	Low	Low	Low
Heise et al. 2023	Low	Low	Low	Low	Low	Low
Johnson et al., 2025	Not applicable	Serious risk (no blinding)	Moderate (ASA24 system but assessors not blinded)	Low	Low	Serious

*Note:* Description: Risk of bias was assessed using the Cochrane RoB 2 tool for the 10 randomized controlled trials, and an adapted ROBINS‐I framework for the two non‐randomized studies, including one cross‐sectional study, with domain‐level judgments applied to ensure consistency across all studies.

Key features of the included studies, including their nutrition components and outcomes, are summarized in Table 2.

**TABLE 2:** Summary of studies evaluating nutrition components alongside incretin therapy for obesity and T2D^a^.

Authors	Title, journal, year	Design and duration	Population	Nutrition component	GLP‐1 RA used	Comparator	Nutrition outcomes	Other outcomes
(1) Structured nutrition interventions								
Schiavo et al. [23]	“Preliminary Evidence Suggests That a 12‐Week Treatment with Tirzepatide Plus Low‐Energy Ketogenic Therapy Is More Effective than Its Combination with a Low‐Calorie Diet in Preserving Fat‐Free Mass, Muscle Strength, and Resting Metabolic Rate in Patients with Obesity”—*Nutrients* 2025	Prospective, non‐randomized comparative study; 12 weeks	*N* = 60 adults with obesity (BMI ≥ 30 kg/m^2^ or ≥ 27 kg/m^2^ with ≥ 1 comorbidity); 30 per group; no T2D; 33 female, 27 male	Two structured, isocaloric diets (~1200 kcal/day), delivered with digital tools and in‐person counseling: ‐ LEKT group: Low‐energy ketogenic therapy with < 30 g/day carbohydrates, 43% protein (1.3 g/kg ideal body weight), 44% fat. Meal plans generated using EatThisMuch app. Nutritional ketosis monitored via blood ketones. ‐ LCD group: Low‐calorie diet with 50% carbohydrate, 20% protein, and 30% fat. Meal plans generated using Nutrigeo 8 software. Participants were not advised to change their physical activity levels during the study.	Tirzepatide, subcutaneous injection: 2.5 mg weekly (4 weeks), then 5 mg weekly (8 weeks)	LEKT vs. LCD, both combined with tirzepatide	‐ Fat mass loss significantly greater in LEKT group (−13.4% ± 2.8%) vs. LCD (−10.2% ± 3.1%); *p* = 0.042 ‐ Fat‐free mass preserved in LEKT (−0.5%) but declined in LCD (−4.3%); *p* = 0.0388 ‐ Muscle strength preserved in LEKT (−0.3%) vs. declined in LCD (−4.1%); *p* = 0.046 ‐ Resting metabolic rate maintained in LEKT (−1.2%) vs. significantly reduced in LCD (−5.3%); *p* = 0.019 ‐ Greater appetite suppression in LEKT group (60%) vs. LCD group (26.7%) ‐ Ketonemia confirmed adherence to LEKT	‐ Similar weight loss in both groups: −10.2% (LEKT) vs. − 9.8% (LCD); *p* = 0.665 ‐ Greater improvements in HbA1c, HOMA‐IR, total cholesterol, and triglycerides in LEKT group ‐ No severe adverse events; mild gastrointestinal symptoms (nausea and constipation, diarrhea, vomiting) occurred at similar rates in both groups
Wadden et al. [28]	“Effect of Subcutaneous Semaglutide vs. Placebo as an Adjunct to Intensive Behavioral Therapy on Body Weight in Adults With Overweight or Obesity: The STEP 3 Randomized Clinical Trial”—*JAMA* 2021	Randomized, double‐blind, placebo‐controlled clinical trial; 68 weeks	*N* = 611 adults with overweight or obesity (BMI ≥ 27 kg/m^2^ with ≥ 1 comorbidity or BMI ≥ 30 kg/m^2^); no T2D; mean age 46 years; 81% female	Structured 68‐week nutrition program: ‐ Weeks 1–8: Low‐calorie diet (1000–1200 kcal/day) using meal replacements (shakes, bars, and portion‐controlled meals supplied by Nutrisystem) ‐ Weeks 9–68: Transition to food‐based hypocaloric diet (1200–1800 kcal/day) based on initial body weight ‐ All participants received 30 individual intensive behavioral therapy sessions with registered dietitians covering calorie goals, nutrition education, physical activity targets (200 min/week), and behavioral change strategies	Subcutaneous semaglutide 2.4 mg once weekly (dose‐escalated from 0.25 mg to 2.4 mg over 16 weeks)	Semaglutide vs. placebo; both groups received identical dietary and behavioral interventions	‐ High adherence to prescribed calorie intake in both groups ‐ Behavioral therapy and structured nutrition support led to reduced energy intake ‐ Transition from meal replacements to food‐based diets was successful ‐ Program designed to support appetite control and long‐term adherence, though exact intake and macronutrient composition not quantified	‐ Mean weight loss: −16.0% (semaglutide) vs. − 5.7% (placebo); *p* < 0.001 ‐ ≥ 5% weight loss: 86.6% (semaglutide) vs. 47.6% (placebo) ‐ ≥ 10%: 75.3% vs. 27.0%; ≥ 15%: 55.8% vs. 13.2% ‐ Greater improvements in waist circumference, systolic BP, HbA1c, fasting insulin, triglycerides, and CRP with semaglutide ‐ GI adverse events (nausea, constipation, and vomiting) more frequent in semaglutide group but generally mild/moderate
Mu et al. [29]	“Efficacy and Safety of Once‐Weekly Semaglutide 2.4 mg for Weight Management in a Predominantly East Asian Population With Overweight or Obesity (STEP 7)”—*The Lancet Diabetes & Endocrinology* 2024	Randomized, double‐blind, multicenter, placebo‐controlled Phase 3a trial; 44 weeks	*N* = 375 adults with overweight or obesity (BMI ≥ 27 kg/m^2^ with ≥ 1 comorbidity or BMI ≥ 30 kg/m^2^); with and without T2D; 77% East Asian participants; mean age ~43 years	Structured lifestyle intervention delivered across 44 weeks: ‐ All participants received counseling on a reduced‐calorie diet (500 kcal/day deficit) and ≥ 150 min/week physical activity, provided by investigators or qualified healthcare professional, either in‐person or by phone ‐ Nutrition plan consisted of a reduced‐calorie diet with a 500 kcal/day deficit calculated from each participant's estimated total energy expenditure ‐ No prescriptive macronutrient composition provided; participants were guided on portion sizes, healthy food choices, and meal planning ‐ Emphasis was placed on sustainable dietary changes and adherence across both arms ‐ Participants also received guidance on increasing physical activity (150 min/week), integrated with dietary goals ‐ Nutrition counseling was reinforced to support adherence and reduce energy intake, but detailed intake or dietary adherence measures were not reported	Subcutaneous semaglutide 2.4 mg once weekly (dose escalation over 16 weeks)	Semaglutide vs. placebo; both groups received identical lifestyle intervention (reduced‐calorie diet and physical activity guidance)	‐ Structured dietary support helped achieve energy intake reductions in both groups though exact dietary intake was not measured ‐ Semaglutide group showed greater weight loss despite identical calorie targets, suggesting appetite suppression and improved adherence ‐ Counseling delivered in person or by phone; frequency not prespecified, no dietary intake and adherence data were collected	‐ Mean bodyweight change: −12.1% (semaglutide) vs. − 3.6% (placebo); *p* < 0.0001 ‐ ≥ 5% weight loss: 85% (semaglutide) vs. 31% (placebo); OR 13.1 ‐ Significant reductions in waist circumference, systolic BP, and lipid profiles (total cholesterol, triglycerides, VLDL) in semaglutide group ‐ Among those with prediabetes, a higher proportion achieved normoglycaemia in the semaglutide group (82% vs. 26%) ‐ Adverse events were predominantly GI‐related and more common in semaglutide group
McGowan et al. [26]	“Efficacy and safety of once‐weekly semaglutide 2.4 mg versus placebo in people with obesity and prediabetes (STEP 10): a randomized, double‐blind, placebo‐controlled, multicenter phase 3 trial”—*The Lancet Diabetes & Endocrinology* 2024	Randomized, double‐blind, placebo‐controlled, multicenter phase 3 trial: 52‐week treatment phase +28‐week off‐treatment follow‐up	*N* = 207 adults with obesity (BMI ≥ 30 kg/m^2^) and prediabetes (HbA1c 6.0%–6.4% or FPG 5.5–6.9 mmol/L); 71% female; 88% White; mean age 53 years; mean BMI 40.1 kg/m^2^	Structured lifestyle program throughout 52‐week treatment phase: ‐ Participants received counseling on a reduced‐calorie diet (500 kcal/day deficit) and ≥ 150 min/week physical activity, provided by dietitians or other healthcare professionals ‐ Guidance on a reduced‐calorie diet with ~500 kcal/day deficit based on estimated energy needs ‐ Counseling on healthy eating patterns, weight loss goals, and sustaining dietary changes ‐ Encouraged ≥ 150 min/week of physical activity ‐ After Week 52, participants received healthy lifestyle counseling per standard care during the 28‐week off‐treatment phase ‐ No standardized meal plans, replacements, or macronutrient prescriptions reported	Subcutaneous semaglutide 2.4 mg once weekly (dose‐escalated over 16 weeks from 0.25 mg)	Placebo with identical lifestyle intervention (reduced‐calorie diet and physical activity counseling)	Quantitative energy or nutrient intake data were not collected.	‐ Clinically meaningful weight loss (−13.9%) with semaglutide and counseling vs. −2.7% with placebo and counseling ‐ 86% of semaglutide group achieved ≥ 5% weight loss vs. 26% with placebo ‐ Sustained reduction in waist circumference: −11.1 cm with semaglutide vs. −2.8 cm with placebo ‐ Partial weight and glycaemic regain occurred after stopping treatment at 52 weeks ‐ HbA1c change: −0.4% with semaglutide vs. +0.1% with placebo ‐ FPG reduction: −0.8 mmol/L vs.−0.2 mmol/L—Systolic BP reduced by −8.8 mmHg vs. −1.0 mmHg ‐ Triglycerides and VLDL cholesterol significantly reduced ‐ Serious adverse events occurred in 9% of both groups; 6% discontinued semaglutide due to AEs (mainly GI‐related)
Anyiam et al. [30]	“Metabolic effects of very‐low calorie diet, Semaglutide, or combination of the two, in individuals with type 2 diabetes mellitus”—*Clinical Nutrition* 2024	Randomized, open‐label, single‐center, three‐arm pilot trial; 12‐week intervention	*N* = 30 adults with T2D mellitus (T2D), aged 18–75 years, BMI 27–50 kg/m^2^; mean age 57 years; 50% female in SEM, 30% in VLCD, 60% in COMB; mean duration of diabetes > 6 years; 10 participants per group (SEM, VLCD, COMB)	‐ VLCD group: Total meal replacement diet (LighterLife); five meal replacements/day (~600 kcal) + up to 200 kcal from fruits/vegetables ‐ COMB group: Identical VLCD protocol alongside semaglutide ‐ SEM group received written dietary advice (portion control, hydration, and fat avoidance) but no structured nutrition support ‐ No dietitian‐led sessions or behavioral counseling in any group ‐ Dietary intake monitored via 4‐day diet diaries at baseline and Week 6 ‐ Macronutrient analysis showed: ‐ Protein % ↑ in VLCD and COMB (to ~31%) ‐ Fat % ↓ in VLCD and COMB (to ~25%) ‐ No significant macronutrient shift in SEM despite reduced energy intake ‐ Strong adherence suggested by energy intake reduction (−1147 kcal VLCD, −1136 kcal COMB, −480 kcal SEM) ‐ No meal pattern, food preference, or satiety data collected	Semaglutide (subcutaneous, once‐weekly): dose escalated from 0.25 mg to 1.0 mg over 4 weeks (accelerated schedule)	Three‐arm comparison: 1. Semaglutide (SEM) 2. Very‐low calorie diet (VLCD) 3. Combination of semaglutide + VLCD (COMB)	‐ Weight loss: VLCD −13.1 kg, COMB −13.5 kg, SEM − 6.0 kg (*p* < 0.01 vs. SEM) ‐ Fat mass loss: VLCD and COMB −9.0 kg vs. SEM −4.0 kg (*p* < 0.01)—Protein intake % energy: VLCD 31.7%, COMB 30.0%, SEM 18.6% (*p* < 0.0001 VLCD/COMB vs. SEM) ‐ Fat intake % energy: VLCD 25.3%, COMB 25.0%, SEM 34.9% (*p* < 0.05 VLCD/COMB vs. SEM) ‐ No change in carbohydrate % energy in any group ‐ Energy intake: significantly reduced in all groups (*p* < 0.05), but largest in VLCD and COMB ‐ COMB group showed synergistic effect: greater beta‐cell function improvement (AIRg ↑ ~ 9×) than either intervention alone ‐ HbA1c reduction greatest in COMB (−20.9 mmol/mol) vs. VLCD (−14.9) and SEM (−9.7); not statistically significant between groups	‐ All groups reduced weight and BMI significantly; largest reductions in VLCD and COMB ‐ HbA1c reduced in all arms; greatest in COMB ‐ Fasting insulin and HOMA‐IR improved in VLCD and COMB only ‐ Lean body mass decreased similarly across groups ‐ AIRg improved significantly in SEM and COMB; COMB increase significantly > VLCD (*p* < 0.01) ‐ Adverse events: ‐ GI side effects (nausea, constipation) most common ‐ One dropout due to Semaglutide intolerance ‐ Six participants contracted COVID‐19; one excluded from follow‐up ‐ No serious safety signals
(2) Lifestyle counseling								
Bliddal et al. [27]	“Once‐Weekly Semaglutide in Persons with Obesity and Knee Osteoarthritis”—*New England Journal of Medicine* 2024	Randomized, double‐blind, placebo‐controlled, multicenter Phase 3 trial: 68‐week treatment period +7‐week follow‐up	*N* = 407 adults with obesity (BMI ≥ 30 kg/m^2^) and moderate‐to‐severe knee osteoarthritis (WOMAC pain score ≥ 40); mean age 56 years; mean BMI 40.3 kg/m^2^; 81.6% female; 60.9% White; conducted at 61 sites across 11 countries	‐ All participants received standardized lifestyle advice on a reduced‐calorie diet and increased physical activity, delivered across 61 international sites ‐ No structured dietary intervention, behavioral counseling, or dietitian involvement reported ‐ Caloric goals not defined; described as general “reduced‐calorie diet” advice ‐ No data reported on dietary intake, macronutrient composition, meal frequency, or satiety ‐ No monitoring of dietary adherence or consistency across countries ‐ Real‐world variation in dietary counseling delivery likely but not evaluated ‐ Nutrition contribution to weight loss unmeasured and cannot be isolated from pharmacological effect	Semaglutide 2.4 mg once weekly (subcutaneous; escalated from 0.25 mg over 16 weeks)	Placebo injection with identical lifestyle advice (diet and physical activity)	No dietary intake or nutrition‐related outcomes were assessed.	‐ Mean weight reduction at 68 weeks: −13.7% (semaglutide) vs.−3.2% (placebo) (*p* < 0.001) ‐ ≥ 5% weight loss: 87.0% (semaglutide) vs. 29.2% (placebo); ≥ 10%: 70.4% vs. 9.2%—Greater reduction in waist circumference: −6.9 cm vs. placebo (*p* < 0.001) ‐ WOMAC pain score change: −41.7 (semaglutide) vs. −27.5 (placebo); difference: −14.1 points (*p* < 0.001) ‐ WOMAC physical function and stiffness scores improved more in semaglutide group ‐ SF‐36 physical function score: +12.0 (semaglutide) vs. 6.5 (placebo) ‐ 6‐min walk test: +56.8 m (semaglutide) vs. +14.2 m (placebo) ‐ Systolic blood pressure reduction: −8 mmHg (semaglutide) vs. none in placebo ‐ Adverse events: mostly gastrointestinal; 6.7% discontinued semaglutide due to AEs ‐ No new safety signals; similar rate of serious adverse events between groups
Aronne et al. [31]	“Continued Treatment With Tirzepatide for Maintenance of Weight Reduction in Adults With Obesity”—*JAMA* 2024	Phase 3, double‐blind, placebo‐controlled, randomized withdrawal trial; 36‐week open‐label lead‐in with tirzepatide followed by 52‐week double‐blind maintenance phase	*N* = 670 adults (mean age 48 years; 70.6% female; mean BMI 38.4 kg/m^2^ at baseline); BMI ≥ 30 or ≥ 27 with ≥ 1 weight‐related complication (excluding T2D); conducted at 70 sites across Argentina, Brazil, Taiwan, and the USA	‐ All participants received lifestyle counseling by qualified professionals throughout the study ‐ Counseling aimed to achieve 500 kcal/day deficit and ≥ 150 min/week of physical activity ‐ No structured dietitian‐led intervention or formal behavioral support program ‐ No reporting of dietary intake, macronutrient composition, meal patterns, or adherence metrics ‐ Dietary and physical activity advice standardized but not monitored or quantified across arms ‐ Effects of lifestyle support vs. pharmacotherapy not isolated; lifestyle assumed consistent between groups	Tirzepatide (subcutaneous, once‐weekly); up‐titrated during lead‐in to maximum tolerated dose (10 or 15 mg); continued at same dose or switched to placebo at Week 36	Continuation of tirzepatide vs. switch to placebo, both with identical lifestyle counseling		‐ Weight loss maintained or increased only with continued tirzepatide:Tirzepatide: −5.5% additional loss from Week 36 to 88 (−25.3% total from baseline)Placebo: +14% regain (net −9.9% from baseline) ‐ 89.5% of tirzepatide group maintained ≥ 80% of prior weight loss vs. 16.6% on placebo (*p* < 0.001) ‐ Waist circumference: −4.3 cm (tirzepatide) vs. +7.8 cm (placebo) ‐ Significant improvements (tirzepatide vs. placebo) from Week 36 to 88:HbA1c, fasting glucose, insulin, lipids, and blood pressureSF‐36 and IWQOL‐Lite‐CT scores (physical and emotional domains) ‐ ≥ 5%, ≥ 10%, ≥ 15%, and ≥ 20% weight loss from Week 0 to 88 significantly more frequent with tirzepatide ‐ ≥ 25% weight loss: 54.5% (tirzepatide) vs. 5.0% (placebo) ‐ Adverse events mostly gastrointestinal (nausea 8.1%, diarrhea 10.7%, vomiting 5.7%) ‐ Serious adverse events similar across groups; 1.8% discontinued tirzepatide due to AEs ‐ Weight regain in placebo group reversed many cardiometabolic benefits
(3) Observational approaches								
Gibbons et al. [13]	“Effects of oral semaglutide on energy intake, food preference, appetite, control of eating and body weight in subjects with type 2 diabetes”—*Diabetes, Obesity and Metabolism*, 2020	Randomized, double‐blind, placebo‐controlled, two‐period cross‐over trial; 12 weeks of oral semaglutide and 12 weeks of placebo, with 5–9 week washout	*N* = 15 adults with T2D (mean age 58.2 years; 86.7% male; mean BMI 30.8 kg/m^2^); stable body weight; treated with diet/exercise or metformin; conducted at a UK clinical site	‐ Ad libitum energy intake assessed during standardized lunch, dinner, and snack box following standard breakfast ‐ Appetite assessed with visual analogue scales (VAS) post‐standard and fat‐rich breakfasts ‐ Control of Eating Questionnaire (CoEQ) used to measure food cravings, hunger, fullness, and eating control ‐ Snack box designed to assess preference for high/low‐fat and sweet/non‐sweet foods ‐ Palatability of meals rated after each meal ‐ Meals prepared and controlled in a clinical research setting; no habitual dietary intake monitored	Oral semaglutide, once‐daily, escalated from 3 mg (Weeks 0–4) to 7 mg (Weeks 4–8), then 14 mg (Weeks 8–12)	Placebo tablets, identical in appearance and administration, followed same dose‐escalation and testing schedule	‐ Total daily ad libitum energy intake ↓38.9% vs. placebo (−5096 kJ; *p* = 0.0001) ‐ Significant ↓ in intake of high‐fat and sweet, and high‐fat foods in snack box ‐ Greater postprandial satiety, fullness, and lower hunger after fat‐rich breakfast (not standard breakfast) ‐ Lower overall appetite scores post‐fat‐rich breakfast (*p* < 0.05) ‐ Improved eating control, fewer cravings on CoEQ vs. placebo ‐ No significant differences in palatability or food aversion (mean VAS > 50 mm for all meals)	‐ Body weight ↓2.7 kg with semaglutide vs. ↓0.1 kg with placebo (mainly due to ↓2.6 kg in fat mass) ‐ No change in lean mass ‐ Fat %age ↓2.0% (semaglutide) vs. ↓0.8% (placebo) ‐ Waist circumference ↓2.4 cm (semaglutide) vs. ↓2.0 cm (placebo), but rebound patterns observed ‐ AEs more common with semaglutide (93 events vs. 51); mostly mild/moderate GI issues ‐ 1 serious AE (MI) in semaglutide group; no deaths; safety consistent with GLP‐1 RA class
Blundell et al. [5]	“Effects of once‐weekly semaglutide on appetite, energy intake, control of eating, food preference and body weight in subjects with obesity”—*Diabetes, Obesity & Metabolism*, 2017	Phase 1, randomized, double‐blind, placebo‐controlled, two‐period crossover trial; 12‐week treatment periods separated by 5‐ to 7‐week washout	*N* = 30 adults with obesity (BMI 30–45 kg/m^2^), no diabetes (HbA1c < 6.5%), mean age 42 years, 66% male, stable weight prior to study; conducted in the United Kingdom	‐ All meals standardized during inpatient assessments; ad libitum lunch, evening meal, and snack box measured over full test day ‐ Food preference measured via Leeds Food Preference Task (LFPT) assessing liking and wanting ‐ Control of eating assessed via Control of Eating Questionnaire (COEQ) ‐ Energy/macronutrient intake measured by weighed food records and digital scales during ad libitum meals ‐ No formal dietary intervention, dietitian contact, or lifestyle counseling ‐ No long‐term follow‐up of dietary behaviors beyond the 12‐week treatment period	Semaglutide (subcutaneous, once‐weekly), escalated to 1.0 mg by Week 9, maintained for final 4 weeks of each treatment period	Placebo injection (matched weekly dosing) in crossover design; participants acted as their own controls	‐ Total ad libitum energy intake over full test day reduced by 24% with semaglutide (−3036 kJ; *p* < 0.0001) ‐ Lunch intake reduced by ~35% (−1255 kJ; *p* < 0.0001); also lower intake at evening meal and snack box ‐ Strong reduction in high‐fat, non‐sweet snack intake (−35%; *p* = 0.0184) ‐ Appetite suppression score higher with semaglutide both fasting and postprandially (*p* = 0.0023) ‐ Lower hunger, greater satiety, reduced food cravings, improved control of eating (COEQ) ‐ LFPT: lower explicit liking and implicit wanting for high‐fat, non‐sweet foods; higher implicit wanting for low‐fat, sweet foods ‐ No differences in palatability ratings or food aversion ‐ No significant change in macronutrient composition of selected foods	‐ Body weight: −5.0 kg with semaglutide vs. +1.0 kg with placebo after 12 weeks ‐ Weight loss largely due to fat mass reduction (3:1 fat to lean mass loss ratio) ‐ Resting metabolic rate (RMR) decreased (−602 kJ/day; *p* = 0.0019), but no difference after lean mass adjustment ‐ No changes in thirst, well‐being, or nausea vs. placebo ‐ No serious AEs; most common were mild/moderate GI symptoms ‐ PK profile as expected; no hypoglycaemic events
Friedrichsen et al. [6]	“The effect of semaglutide 2.4 mg once weekly on energy intake, appetite, control of eating, and gastric emptying in adults with obesity”—*Diabetes, Obesity & Metabolism*, 2021	Phase 1, randomized, double‐blind, placebo‐controlled, parallel‐group trial; 20‐week intervention with 7‐week follow‐up	*N* = 72 adults with obesity (mean age 42.8 years; 61.1% male; mean BMI 34.4 kg/m^2^; mean weight 105.5 kg); adults aged 18–65 years; BMI 30–45 kg/m^2^; no recent weight change; excluded diabetes, GI disorders, or recent medication use affecting weight	‐ Participants consumed a standardized breakfast before ad libitum lunch test ‐ No structured dietary counseling or nutritionist‐led behavioral intervention ‐ Ad libitum energy intake measured at baseline and Week 20 using a standardized excess meal ‐ Appetite assessed via VAS and control of eating via the CoEQ ‐ Meals provided by study site; no monitoring of diet outside assessment periods ‐ No formal tracking of macronutrient composition beyond standard meal description	Semaglutide 2.4 mg once‐weekly subcutaneous injection; 16‐week dose escalation (0.25 → 0.5 → 1.0 → 1.7 → 2.4 mg) followed by 5 weeks at 2.4 mg	Placebo injection with matched dosing schedule and blinding	‐ 35% lower ad libitum energy intake at Week 20 in semaglutide group (−940 kJ; *p* < 0.0001)—Appetite VAS: lower hunger and prospective food consumption, higher satiety and fullness with semaglutide vs. placebo (all *p* < 0.02) ‐ Appetite suppression score significantly higher with semaglutide (ETD: +13 mm; *p* = 0.001) ‐ CoEQ: less hunger, better control of eating, fewer cravings for savory and dairy foods (*p* < 0.05) ‐ Energy intake measured at a single ad libitum lunch; no habitual dietary data.	‐ Body weight reduced by 10.4 kg (−9.9%) with semaglutide vs. 0.4 kg (−0.4%) with placebo over 20 weeks ‐ No evidence of delayed gastric emptying based on paracetamol absorption (AUC₀‐₅h increased 8% but non‐significant after adjusting for body weight) ‐ No difference in paracetamol Cmax or tmax ‐ Safety profile consistent with previous GLP‐1 RA trials; GI events more common with semaglutide but mostly mild ‐ Study powered to detect change in gastric emptying but revealed strong secondary effects on appetite, control of eating, and body weight
Heise et al. [14]	“Tirzepatide Reduces Appetite, Energy Intake, and Fat Mass in People With Type 2 Diabetes”—*Diabetes Care*, 2023	Secondary analysis of a phase 1, randomized, double‐blind, parallel‐arm trial; 28‐week duration; comparisons among tirzepatide 15 mg, semaglutide 1 mg, and placebo	*N* = 117 participants with T2D (mean age 61.1 years tirzepatide group; 68.9% male; mean BMI 31.3 kg/m^2^); conducted across multiple centers; inclusion required T2D diagnosis; modest differences in age and diabetes duration across groups	‐ Ad libitum buffet‐style lunch conducted at baseline, weeks 8, 16, and 28 ‐ Energy intake measured at each timepoint ‐ Appetite assessed via fasting VAS ratings (hunger, fullness, satiety, prospective consumption) ‐ Composite appetite score calculated ‐ No structured dietary advice, counseling, or behavioral intervention ‐ No 24‐h dietary intake assessed ‐ Nutrition assessments limited to lunch; total daily intake, macronutrient composition, and dietary adherence not evaluated	Tirzepatide 15 mg (once weekly, subcutaneous), compared with semaglutide 1 mg (once weekly, subcutaneous; lower than the 2.4 mg dose used for obesity management)	Placebo and semaglutide 1 mg; randomization ratio 3:3:2 (tirzepatide: *N* = 45; semaglutide: *N* = 44; placebo: *N* = 28)	‐ At Week 28, energy intake during lunch reduced by 309.8 kcal with tirzepatide vs. placebo (*p* < 0.001) ‐ Appetite VAS scores significantly improved with tirzepatide and semaglutide vs. placebo; no significant difference between tirzepatide and semaglutide ‐ Fasting appetite composite scores increased (indicating reduced appetite); satiety and fullness increased, hunger and prospective consumption decreased ‐ No significant difference in lunch energy intake between tirzepatide and semaglutide (−64.3 kcal; *p* = 0.187)	‐ Tirzepatide reduced body weight by −11.2 kg at Week 28 vs. placebo (*p* < 0.001), vs. −7.0 kg with semaglutide ‐ Fat mass loss greater with tirzepatide vs. semaglutide (−3.8 kg difference; *p* = 0.002) ‐ Reductions in fat‐free mass also observed but smaller ‐ Weight loss mainly driven by fat mass reduction ‐ No structured metabolic biomarker analysis reported ‐ Safety outcomes reported in separate publication; AEs consistent with incretin class; no new safety signals
Johnson et al. [20]	“Investigating nutrient intake during use of glucagon‐like peptide‐1 receptor agonist: a cross‐sectional study”—*Frontiers in Nutrition*, 2025	Cross‐sectional observational study: 3‐day dietary intake collected via ASA24 food record; single‐timepoint data collection	Adults (*N* = 69), 79.7% female, recruited in the US via online platform; current users of GLP‐1RA for ≥ 1 month; liraglutide users excluded due to low sample (*n* = 1)	Three‐day food records analyzed for energy, macronutrients, and micronutrients; intake compared against DRI; MyPlate food group servings also assessed	Semaglutide (53.6%) Tirzepatide (33.3%) Dulaglutide (11.6%) Liraglutide (*n* = 1) excluded from main analysis	No formal comparator group; internal comparisons by medication type, duration, and self‐reported changes in food intake	‐ 72% consumed energy below estimated needs ‐ 54% consumed protein < 0.8 g/kg/day; 43% met ≥ 1.2 g/kg/day, only 10% ≥ 1.6 g/kg/day ‐ Mean intake significantly below DRI for fiber (14.5 g), calcium (863 mg), iron (12.1 mg), magnesium (266 mg), potassium (2186 mg), vitamin A (560 mcg), vitamin C (51 mg), vitamin D (4 mcg), vitamin E (9.6 mg), and choline (305 mg) ‐ 63%–99% of participants below DRI for multiple micronutrients; highest deficits for vitamin D (98.6%), potassium (98.6%), and choline (94.2%) ‐ Participants overconsumed % calories from fat (mean 39.9%) and saturated fat (26 g) ‐ MyPlate servings significantly below guidelines for fruit, vegetables, grains, and dairy (*p* < 0.01)	‐ Most common side effects: nausea (53.7%), diarrhea (27.8%), and fatigue (30.3%) ‐ 76% reported appetite change since starting GLP‐1 RA ‐ 49% did not receive advice on managing side effects; only 20% referred to an RDN ‐ No data collected on weight loss, lean mass, or biochemical outcomes

*Note:* Units and symbols: % (percent), + (plus/with), < (less than), ≤ (less than or equal to), ≥ (greater than or equal to), → (dose escalation), ~ (approximately), cm (centimeters), d (day), g (grams), g/kg/day (grams per kilogram per day), kg (kilograms), kg/m^2^ (kilograms per square meter), kJ (kilojoules), kJ/day (kilojoules per day), m (meters), mm (millimeters), mm Hg (millimeters of mercury), mmol/L (millimoles per liter), mmol/mol (millimoles per mole), min/wk. (minutes per week), kcal/day (kilocalories per day).

Abbreviations: AE (adverse event), AIRg (Acute Insulin Response to Glucose), ASA24 (Automated Self‐Administered 24‐hour Dietary Recall), BP (blood pressure), CHO (carbohydrate), CoEQ (Control of Eating Questionnaire), COMB (Combination group: VLCD + Semaglutide), CRP (C‐reactive protein), DRI (Dietary Reference Intake), ETD (Estimated Treatment Difference), F (fat), FFM (fat‐free mass), FM (fat mass), FPG (fasting plasma glucose), GI (gastrointestinal), GLP‐1 RA (Glucagon‐Like Peptide‐1 Receptor Agonist), HbA1c (glycated hemoglobin), HOMA‐IR (Homeostatic Model Assessment of Insulin Resistance), IWQOL‐Lite‐CT (Impact of Weight on Quality of Life‐Lite Clinical Trials version), kcal (Kilocalories), LCD (Low‐Calorie Diet), LEKT (Low‐Energy Ketogenic Therapy), LFPT (Leeds Food Preference Task), OA (osteoarthritis), OR (odds ratio), P (protein), PK (pharmacokinetics), RCT (randomized controlled trial), RDN (Registered Dietitian Nutritionist), RMR (resting metabolic rate), SD (standard deviation), SEM (semaglutide group), SF‐36 (Short Form‐36 Health Survey), s.e.m. (standard error of the mean), T2D (Type 2 diabetes), VAS (Visual Analogue Scale), VLCD (very‐low‐calorie diet), VLDL (very‐low‐density lipoprotein), WC (waist circumference), wk. (week), WOMAC (Western Ontario and McMaster Universities Osteoarthritis Index), AUC (Area Under the Curve), Cmax (maximum concentration), tmax (time to maximum concentration), *N* (sample size).

^a^ Overview of included trials examining energy intake, appetite, and nutrition‐related effects in adults receiving GLP‐1 RAs, with or without structured dietary intervention. Dietitian involvement was minimal or absent unless explicitly reported in the “Nutrition Component” column. Studies include heterogeneity and may include clinical trials of incretin therapies as well as secondary analyses, and observational studies.

### 3.1 Structured Dietary Protocols

Five studies employed structured nutrition protocols alongside incretin therapy. These included VLCD, ketogenic approaches, or defined hypocaloric plans with specific macronutrient targets [23, 26, 28, 29, 30].

In a non‐randomized, high‐risk comparative study, Schiavo et al. (2025) evaluated tirzepatide, initiated at 2.5 mg and escalated to 10 mg then 15 mg once weekly, combined with either a low‐energy ketogenic therapy (LEKT; approximately 1200 kcal per day, less than 30 g of carbohydrate, 43% protein, and 44% fat) or a balanced low‐calorie diet (LCD; 50% carbohydrate, 20% protein, and 30% fat) in 60 adults living with obesity [23]. Both groups received digitally supported meal planning and in‐person counseling delivered by certified nutritionists, not registered dietitians. The LEKT group achieved significantly greater fat mass reduction (13.4% compared with 10.2%; *p* = 0.042) and better preservation of fat‐free mass (0.5% compared with 4.3%; *p* = 0.039), muscle strength (0.3% compared with 4.1%; *p* = 0.046), and resting metabolic rate (1.2% compared with 5.3%; *p* = 0.019). Appetite suppression occurred more frequently in the LEKT group, at 60% compared with 27%, despite similar total weight loss of approximately 10% [23].

In a 68‐week double‐blind RCT, Wadden et al. (2021) investigated semaglutide at 2.4 mg once weekly in combination with intensive behavioral therapy [28]. This included a structured meal replacement plan followed by a hypocaloric food‐based diet. Participants attended 30 sessions led by registered dietitians. Energy intake was initially restricted to 1000 to 1200 kcal per day (kcal/day), gradually increasing to 1200 to 1800 kcal depending on weight change, alongside physical activity and behavioral counseling. Dietary intake was not quantitatively assessed, but weight loss was significantly greater with semaglutide compared with placebo (16.0% compared with 5.7%; *p* < 0.001), and adherence to calorie targets was reported to be high [28].

Mu et al. (2024) conducted a 44‐week multicenter randomized trial in East Asian adults living with obesity or overweight, with or without T2D. Participants received semaglutide at 2.4 mg once weekly or placebo, combined with a 500 kcal/day energy deficit and physical activity targets [29]. Participants received counseling on a reduced‐calorie diet and physical activity from investigators or other qualified healthcare professionals, delivered in person or by telephone; frequency was not prespecified. No macronutrient prescription was specified, but participants received guidance on portion control, meal planning, and healthy food choices. Dietary intake was not measured. However, weight loss was significantly greater with semaglutide (12.1% compared with 3.6%; *p* < 0.0001), and 85% achieved at least 5% weight loss [29].

In a 52‐week trial, McGowan et al. (2024) evaluated semaglutide at 2.4 mg once weekly combined with a structured lifestyle intervention in adults living with obesity and prediabetes [26]. Participants received lifestyle counseling on a reduced‐calorie diet and physical activity from dietitians or other healthcare professionals, targeting a 500 kcal/day energy deficit. Macronutrient targets and dietary intake data were not reported. Weight loss with semaglutide was greater than with placebo (13.9% compared with 2.7%), and 81% of participants reverted to normoglycemia compared with 14% in the placebo group [26].

In a three‐arm pilot trial, Anyiam et al. (2024) compared semaglutide at 1.0 mg once weekly alone, a food‐based VLCD alone (approximately 800 kcal/day), or VLCD plus semaglutide in 30 adults living with T2D [30]. All groups received written dietary guidance, but no registered dietitian involvement or structured counseling. The combination group achieved greater fat mass loss (9.0 kg compared with 4.0 kg), greater reductions in energy intake (1136 kcal/day compared with 480 kcal/day), and a higher percentage of energy from protein (30.0% compared with 18.6%) than the semaglutide‐alone group. Lean mass declined similarly across all arms. Improvements in beta‐cell function and insulin sensitivity were greatest in the combination group [30].

### 3.2 Lifestyle Counseling

Two large RCTs incorporated semaglutide or tirzepatide into broader lifestyle programs that did not include specific dietary prescriptions [27, 31]. In the STEP 9 trial, Bliddal et al. (2024) evaluated semaglutide at 2.4 mg once weekly in adults living with overweight or obesity and knee osteoarthritis [27]. Participants across 61 sites in 11 countries received general advice to follow a reduced‐energy diet and increase physical activity, but there were no structured meal plans, dietitian‐led sessions, or behavioral nutrition interventions. Nutrition protocols and dietary adherence were not reported. Despite the absence of formal dietary support, semaglutide led to significantly greater weight loss compared with placebo (13.7% compared with 3.2%; *p* < 0.001). Additional improvements were observed in pain, physical function, and walking distance [27].

The SURMOUNT‐4 trial assessed tirzepatide initiated at 2.5 mg and escalated to 10 mg then 15 mg once weekly during a 36‐week open‐label lead‐in phase, after which participants were randomized to continue tirzepatide or switch to placebo for a further 52 weeks [31]. All participants received lifestyle counseling from qualified healthcare professionals. This included advice to achieve a 500 kcal/day energy deficit and engage in at least 150 min of physical activity per week. However, no structured dietary intervention was delivered, there was no involvement of registered dietitians, and no information was provided on nutritional intake or adherence. During the maintenance phase, those who switched to placebo regained weight (mean increase of 14% between weeks 36 and 88), while those who continued tirzepatide lost an additional 5.5% of body weight. This resulted in a total weight loss of 25.3% from baseline. The role of dietary change in these outcomes was not evaluated [31].

### 3.3 Observational Approaches

Five mechanistic or observational studies assessed energy intake, appetite, food preferences, or habitual dietary intake in individuals receiving semaglutide or tirzepatide, without structured nutrition interventions or dietitian support [5, 6, 13, 14, 20]. Four studies were conducted in controlled experimental settings [5, 6, 13, 14], while one provided real‐world dietary data through cross‐sectional self‐report [20]. Using a randomized crossover design, one study evaluated the effects of semaglutide at 1.0 mg once weekly on food intake and appetite responses in 30 adults with obesity [5]. Total energy intake declined by 24%, with a reduction of 3036 kJ per day (kJ/day) across ad libitum test meals. Appetite scores measured by visual analogue scales indicated reduced hunger and increased satiety. Weight loss was primarily attributed to fat mass, with a fat‐to‐lean mass loss ratio of approximately 3 to 1 [5].

Another trial investigated oral semaglutide, titrated to 14 mg daily, in 15 adults with T2D [13]. Participants showed a 38.9% reduction in total energy intake compared with placebo. Appetite regulation was reported to improve, including enhanced satiety and reduced food cravings, as measured by the Control of Eating Questionnaire [13]. No significant changes in meal palatability were observed, and fat mass reduction accounted for most of the observed weight loss [13]. In a parallel‐group randomized trial of 72 adults with obesity, participants receiving semaglutide at 2.4 mg once weekly experienced a 35% lower energy intake than placebo at Week 20 [6]. Appetite scores improved across multiple domains, including hunger, satiety, and control of eating, without affecting palatability or gastric emptying. Body weight decreased by 10.4 kg in the semaglutide group compared with 0.4 kg in the placebo group [6].

A 28‐week analysis compared tirzepatide at 15 mg once weekly with semaglutide at 1.0 mg and placebo in 117 adults living with T2D [14]. At a buffet‐style meal, tirzepatide reduced ad‐libitum energy intake by 309.8 kcal relative to placebo. Both tirzepatide and semaglutide improved appetite scores, with lower hunger and greater post‐meal fullness. Fat mass loss was significantly greater with tirzepatide, although declines in lean mass/fat free mass were also noted. No structured dietary counseling was provided [14].

In a cross‐sectional study of 69 adults using GLP‐1 RAs, most of whom were taking semaglutide (53.6%) or tirzepatide (33.3%) at clinically approved doses, participants completed three‐day food records to assess nutrient intake [20]. Results revealed widespread nutrient inadequacies, with 72% consuming less than their estimated energy needs and over 90% failing to meet dietary reference intakes for vitamin D, potassium, and choline. Only 10% met protein intakes of at least 1.6 g per kilogram per day [20], and fewer than half had received any dietary advice [20]. The study underscores potential risks of nutrient inadequacies during incretin therapy in real‐world settings and highlights a lack of routine dietitian involvement [20].

## 4 Discussion

This systematic scoping review aimed to synthesize and critically appraise the current evidence on nutrition components, including structured dietary protocols, lifestyle counseling, and observational assessments incorporated into clinical trials and other study designs examining next‐generation incretin therapies, specifically semaglutide and tirzepatide, in adults living with obesity or T2D. Across the 12 included studies, these medications were consistently associated with appetite suppression, reductions in ad libitum energy intake, and substantial fat mass loss. However, the trials varied widely in their design, dietary delivery models, and approaches to nutritional assessment. Differences in energy prescriptions, intervention intensity, outcome measurement tools, and the involvement of registered dietitians or other qualified professionals introduced considerable heterogeneity, limiting the ability to isolate the individual or combined effects of pharmacological therapy and nutritional support. Only three studies involved dietitians or similarly trained nutrition professionals, and even in these cases, nutrition‐related outcomes were not consistently or comprehensively assessed. The cross‐sectional study by Johnson et al. (2025) provided additional insights into real‐world dietary practices among incretin therapy users, revealing widespread nutrient inadequacies, insufficient protein intake, and minimal access to professional dietary advice [20]. These findings highlight the potential for nutritional complications outside of trial settings and underscore the need for routine, dietitian‐led support during incretin therapy, ideally as part of holistic multidisciplinary care.

### 4.1 Structured Dietary Protocols

Across trials, validated measures of dietary quality were not used, representing a key evidence gap. Structured dietary protocols offered the clearest evidence regarding the nutritional effects of combining defined dietary support with GLP‐1 RA therapy. Trials that used specific energy prescriptions and macronutrient targets, particularly those implementing VLCD or ketogenic regimens, demonstrated greater preservation of lean mass and resting metabolic rate compared with balanced low‐calorie diets or semaglutide alone [23]. Higher protein intake was associated with reduced lean mass loss, even when total weight loss was equivalent [23].

The trial by Wadden et al. [28], which included 30 sessions delivered by registered dietitians, presented a model of intensive, long‐term behavioral and dietary support. However, no quantitative assessments of dietary intake or biochemical measures of nutrient adequacy were reported. Across all structured interventions [23, 26, 28, 29], the lack of diet‐only comparator arms and the inconsistent measurement of protein intake and micronutrient deficiency limited conclusions about the independent contribution of the nutritional component and its role in mitigating nutritional risks.

Nevertheless, the evidence suggests that macronutrient composition and structured delivery of the dietary interventions may influence outcomes beyond weight loss alone, including the preservation of metabolic rate and muscle mass and possibly satiety and adherence.

### 4.2 Lifestyle Counseling

The trials that incorporated lifestyle counseling contributed large sample sizes and offered pragmatic relevance to real‐world clinical practice. However, their interpretability was constrained by the absence of standardized nutrition protocols and a lack of reporting on dietary adherence, nutrient intake, or nutritional sufficiency. Involvement of registered dietitians was minimal or absent, and no trial collected comprehensive dietary intake data or assessed nutrition‐related complications such as vitamin or mineral deficiencies.

Although significant weight loss was observed with incretin therapies in these settings [26, 27], it remains uncertain whether lifestyle counseling contributed meaningfully to these outcomes. The absence of nutritional endpoints such as protein intake, micronutrient intake, or changes in body composition limits the ability to assess the broader impact of nutrition within these interventions.

These trial designs align with routine clinical practice, such as primary care and commercial weight management services [32, 33], but offer limited insight into how best to integrate nutritional care with pharmacotherapy. They highlight a critical evidence gap in determining whether general dietary advice is adequate or whether structured, dietitian‐led support can provide additional clinical benefits and minimize risk.

### 4.3 Observational Approaches

The observational studies embedded within clinical trials and those using other study designs provided important insights into appetite regulation and habitual dietary intake during incretin therapy, despite the absence of structured nutrition interventions or dietitian support [5, 6, 13, 14, 20]. Experimental trials using standardized test meals consistently demonstrated substantial reductions in ad‐libitum energy intake (24%–39%) and improvements in satiety and control of eating under semaglutide or tirzepatide [5, 6, 13, 14]. Despite lean mass loss being observed across studies, no randomized trial monitored habitual nutrient intake. Only the cross‐sectional study collected 3‐day diet diary records and compared intakes with DRIs and MyPlate targets [20], which highlighted significant macronutrient and micronutrient deficiencies, including suboptimal protein intake in real‐world incretin therapy users, most of whom had not received nutrition or dietetic support alongside the drugs. Together, these findings raise concerns about undetected nutritional risks during pharmacotherapy and underscore the need for dietary monitoring in both research and particularly in clinical practice with their rapid adoption in obesity management.

## 5 Implications for Clinical Practice and Policy

Although semaglutide and tirzepatide are highly effective for weight loss, there remains limited evidence from clinical trials to guide optimal nutritional care during therapy. Most studies did not include dietitian‐led support or comprehensive dietary monitoring, and recent cross‐sectional data have highlighted widespread nutrient inadequacies among real‐world GLP‐1 RA users [20].

Based on the available evidence, routine dietitian involvement should be prioritized from the start of treatment to provide a tailored nutritional assessment and individualized support. Clinicians should ideally consider recommending nutrient‐dense, high‐quality protein intake of 1.2–1.5 g/kg/day based on adjusted body weight and distributed evenly across meals to minimize lean mass loss, which aligns with guidelines for surgical weight loss patients and nutritional priorities to support GLP‐1 therapy for obesity [10, 34]. Individualisation is required for patients with renal impairment [35]. While specific macronutrient prescriptions were not consistently tested across trials, higher protein intake appeared more protective than either low‐fat or balanced macronutrient approaches [23]. Nonetheless, if gastrointestinal side effects such as steatorrhoea are present, moderate fat reduction may be considered on an individual basis [12].

There is insufficient trial evidence to recommend strict low‐fat diets while using incretin therapy, but overconsumption of total and saturated fat was recorded in observational studies [20], suggesting there might be a need for individualized fat intake guidance, aligned with broad national recommendations such as the UK Eatwell Guide [36] and the US Dietary Guidelines for Americans 2020–2025 [37], and refined by bariatric‐specific targets from the Allied Health Nutritional Guidelines for the Surgical Weight Loss Patient [34]. Similarly, although meal frequency was not explicitly tested in most trials, strategies such as small, frequent meals may help reduce common side effects such as nausea and improve tolerability [12], especially in the early stages of treatment.

Baseline and periodic biochemical monitoring for micronutrient deficiencies (e.g., vitamin B12, folate, vitamin D, iron, and thiamine) is warranted, informed by existing bariatric care models [38]. Where applicable, psychological support should be offered, targeting emotional and disinhibited eating and other individualized care, with mindfulness‐based strategies showing promise [39].

At a systems level, healthcare services must integrate dietitians and the wider MDT into incretin therapies prescribing pathways, and reimbursement policies should reflect the need for comprehensive nutritional and behavioral care to support safe, equitable, and sustainable outcomes. At present, this remains missing from the standard of care and concerns continue to mount regarding clinical services prescribing incretin therapies without comprehensive wrap‐around care, which should be mandatory.

## 6 Limitations of the Evidence Base

Despite the increasing use of incretin therapies in obesity and T2D management, the literature combining these agents with structured nutrition interventions remains limited and methodologically diverse. Only 12 studies addressed both components, most enrolling fewer than 120 participants and with follow‐up periods ranging from 12 to 68 weeks. These constraints limit statistical power and preclude robust evaluation of long‐term outcomes, including sustained weight maintenance, micronutrient status, and preservation of lean tissue mass [5, 18, 28]. The dietary approaches varied widely, from VLC ketogenic regimens to unstructured ad libitum feeding assessments. In addition, there was inconsistent use of validated dietary assessment tools and variation in adherence to recommended macronutrient targets or dietary quality, reducing the comparability and generalizability of findings across trials [13, 14].

The majority of trials prioritized weight and glycaemic endpoints such as glycated hemoglobin (HbA1c) and insulin resistance (i.e., HOMA‐IR), while broader nutritional outcomes were frequently underreported. Key variables such as dietary protein intake, micronutrient sufficiency, and gastrointestinal tolerability were seldom measured, despite known risks of nutritional deficiencies and cholelithiasis during rapid weight loss [38, 40, 41, 42, 43]. Moreover, most studies assessed either “lean mass” using dual‐energy X‐ray absorptiometry (DEXA) or “fat free mass” via bioelectrical impedance analysis (BIA), which cannot differentiate skeletal muscle from other lean tissue components including organs and extracellular fluid [44]. These are estimates derived from indirect calculation rather than direct measurement [45]. This limits interpretation of whether reported reductions reflect true muscle mass loss. More accurate imaging modalities such as MRI, which provide better specificity for muscle tissue, were not employed in any of the included studies.

Few studies involved registered dietitians or other qualified professionals in the design or delivery of the nutrition intervention, further reducing the reliability of reported dietary adherence and intake data and presenting concern about dietary adequacy while on these therapies. Finally, restrictive eligibility criteria excluded many high‐risk groups, such as older adults, individuals with sarcopenic obesity, ethnically diverse populations, or those with multimorbidity. These exclusions limit the applicability of findings to the real‐world population most vulnerable to lean tissue loss and micronutrient insufficiency [20].

Notably, most included trials recruited high‐income populations, limiting generalizability. In real‐world settings, obesity disproportionately affects those in low‐income communities with higher levels of food insecurity [46, 47, 48]. This may lead to poorer dietary quality [49], amplify nutritional risks, and highlight the importance of accessible, dietitian‐led care. Future research must incorporate social determinants of health to ensure guidance is equitable.

## 7 Future Directions

To strengthen the evidence base for nutritional management in people treated with incretin therapies, future trials should adopt factorial randomized designs that isolate the independent and combined effects of pharmacological and dietary interventions. Emerging incretin therapies, including triple agonists such as retatrutide, are anticipated to achieve even greater weight loss than semaglutide or tirzepatide [50, 51], which further strengthens the need to ensure nutritional adequacy and greater dietitian involvement as pharmacological efficacy. Large‐scale, long‐term studies are needed to compare, at minimum: incretin therapy combined with high‐protein, nutritionally adequate hypocaloric diets; incretin therapy with balanced hypocaloric diets; diet‐only control arms; and placebo with usual care. Study populations should be stratified by age, sex, ethnicity, comorbidities including sarcopenic obesity, and care setting to maximize external validity.

Dietary interventions should be standardized across trials with consideration of using established dietary patterns such as the Mediterranean, Dietary Approaches to Stop Hypertension (DASH), Nordic, high‐protein, or plant‐based diets, tailored to local food systems and guided by emerging international frameworks and guidelines, to optimize cardiometabolic and nutritional outcomes [7, 52, 53]. Recent expert guidance from the US encourages patient‐centered initiation of GLP‐1s, baseline screening (i.e., dietary habits, emotional triggers, disordered eating, and relevant medical conditions), comprehensive exams (i.e., muscle strength, function, and body composition), social determinants of health screening, lifestyle assessment (i.e., aerobic activity, strength training, sleep, mental stress, substance use, and social connections), and dietitian support (i.e., counseling, group‐based visits, telehealth, and digital platforms) [10], but more evidence from carefully designed trials is needed.

Outcome measures must extend beyond weight change to include accurate body composition assessment. While DEXA and BIA are common, future studies should consider incorporating MRI, where feasible, to more precisely quantify changes in muscle mass [54, 55] However, due to limitations of access to more comprehensive body composition measures in clinical practice, the use of BIA offers a practical solution to understanding the impact of incretin therapies on, as a minimum, fat free mass.

Additional core endpoints should include biomarkers of protein status, full micronutrient panels (including iron, vitamin B12, folate, vitamin D, calcium, and thiamine), and gallbladder imaging to monitor for cholelithiasis in symptomatic individuals [41], consistent with bariatric surgery protocols [38, 42, 43]. Evaluation of metabolic parameters such as HOMA‐IR, resting metabolic rate, and appetite‐regulating hormone profiles would further clarify the mechanistic interactions between diet and incretin therapy [7].

To support real‐world implementation, trials should integrate delivery models that include dietitian‐led counseling, digital care including dietary monitoring, and structured nutrition protocols aligned with bariatric care guidance [38]. Digital, potentially hybrid, interventions offer a unique opportunity to allow wider access and monitoring of patients for nutritional risk while on incretin therapies [56]. Although some health‐economic analyses have examined incretin therapies [44], few studies have evaluated the cost‐effectiveness of combined nutritional support. Future trials should include embedded economic evaluations measuring cost per quality‐adjusted life year (QALY), changes in healthcare utilization, and cost savings from prevention of nutrition‐related complications.

## 8 Conclusion

The integration of dietary protocols, lifestyle counseling, and observational assessments into next‐generation incretin therapy programs presents an important opportunity to enhance weight loss efficacy while also supporting lean mass preservation and potential macro‐ and micronutrient deficiencies. Although current trials consistently demonstrate that semaglutide and tirzepatide lead to substantial appetite suppression and fat mass reduction, the wide variation in dietary protocols, lack of standardized nutrition assessments, and limited early involvement of registered dietitians reduce the applicability of findings to clinical practice. Until high‐quality factorial RCTs establish optimal dietary strategies, clinicians should adopt a holistic multidisciplinary, collaborative, and patient‐centered approach that includes nutritional expertise alongside medical and behavioral care. Dietitians should be engaged from the outset to deliver individualized dietary advice to manage common gastrointestinal side effects and ensure adequate intake of key vitamins and minerals through dietary counseling and supplementation where needed. Only through such interdisciplinary, rigorously designed research can evidence‐based nutrition guidelines be developed to support optimal outcomes in people using next‐generation incretin therapies.

## Funding

This work was supported by the National Institute for Health and Care Research (303041).

## Conflicts of Interest

M.S. and C.F.R. declare no competing interests. A.B. declares researcher‐led grants from the National Institute for Health Research, Rosetrees Trust, MRC, INNOVATE UK, British Dietetic Association, British Association of Parenteral and Enteral Nutrition, BBSRC, the Office of Health Improvement and Disparities and NovoNordisk. A.B. reports honoraria from Novo Nordisk and Eli Lilly outside the submitted work and is on the Medical Advisory Board and a shareholder of Reset Health Clinics Ltd.

## Supporting information


**Table S1: Supplementary** PICOS eligibility criteria for study inclusion
**Table S2: Supplementary**. PICOS Framework for Included Studies

## Data Availability

Data sharing not applicable to this article as no datasets were generated or analysed during the current study.
