# Supplementary material for: Nutrition Strategies for Next‐Generation Incretin Therapies: A Systematic Scoping Review of the Current Evidence
Source: Obes Rev. 2026 Jan 7;27(6):e70079. doi: 10.1111/obr.70079 (PMC13136787; doi:10.1111/obr.70079)
Supplement: Supplementary file 1 — Table S1: Supplementary PICOS eligibility criteria for study inclusion Table S2: Supplementary. PICOS Framework for Included Studies [file OBR-27-e70079-s001.pdf]

Supplementary Materials

Supplementary Table S1. PICOS eligibility criteria for study inclusion

Element	Criteria
**P**	Adults (≥ 18 years) with obesity (BMI ≥ 30 or ≥ 27 + comorbidity) or T2D receiving semaglutide or tirzepatide
**I**	Any nutrition component alongside GLP-1 RA therapy (e.g. hypocaloric diets, ketogenic protocols, meal replacements, meal provision, counselling)
**C**	Placebo, GLP-1 RA without nutrition components, or diet alone; for observational studies, internal comparisons (e.g. dose, duration, reported dietary intake)
**O**	Energy intake, body composition (fat/lean mass), protein adequacy, micronutrient status, patient-reported dietary experience
**S**	Randomised controlled trials, prospective comparative studies, and cross-sectional studies published January 2015 - April 2025

Abbreviations: BMI (Body Mass Index), C (Comparator), e.g. (for example), GLP-1 RA (Glucagon-Like Peptide-1 Receptor Agonist), I (Intervention), O (Outcome), P (Population), PICOS (Population, Intervention, Comparator, Outcome, Study Design), S (Study Design), T2D (Type 2 Diabetes).
Units and symbols: ≥ (Greater than or equal to), + (Plus).

Supplementary Table S2. PICOS Framework for Included Studies

Study ID	Population (P)	Intervention (I)	Comparator (C)	Outcomes (O)	Study Design (S)
Schiavo et al. 2024	Adults with obesity (BMI ≥ 30 kg/m² or ≥ 27 kg/m² + comorbidity)	Tirzepatide (2.5→5 mg wkly) + low-energy ketogenic therapy (~1200 kcal/d, < 30 g CHO, 43 % P)	Tirzepatide + balanced low-calorie diet (~1200 kcal/d, 50 % CHO)	Energy intake, body composition (FM, FFM), RMR, MS, appetite/satiety scores, glycaemia	Prospective, non-randomised comparative (12 wk)
Wadden et al. 2021, STEP 3	Adults with overweight/obesity (BMI ≥ 27 + comorbidity or ≥ 30)	Semaglutide 2.4 mg wkly + intensive behavioral therapy (meal replacements → hypocaloric diet)	Placebo + identical behavioral therapy/diet	% weight change, energy intake patterns, GI AEs, waist circumference	RCT, parallel, double-blind (68 wk + 7 wk follow-up)
Mu et al. 2024, STEP 7	East Asian adults with overweight/obesity (BMI ≥ 27 or ≥ 30, ± T2D)	Semaglutide 2.4 mg wkly + −500 kcal/d diet + 150 min/wk activity	Placebo + identical lifestyle intervention	% weight change, energy intake, glycaemia, lipids, WC, BP	RCT, parallel, double-blind, multicentre (44 wk)
McGowan et al. 2024, STEP 10	Adults with obesity + prediabetes (BMI ≥ 30 or ≥ 27)	Semaglutide 2.4 mg wkly + −500 kcal/d diet + activity + dietitian counselling	Placebo + identical lifestyle intervention	% weight change, normoglycaemia rates, glycaemia, lipids, WC, BP	RCT, parallel, double-blind, multicentre (52 wk + 28 wk off)
Anyiam et al. 2024	Adults with T2D (BMI 27-50)	Semaglutide 0.25→1 mg wkly + 800 kcal/d VLCD	Semaglutide alone (identical counselling)	Energy intake, FM, FFM, insulin sensitivity (HOMA-IR), AIRg, glycaemia	Pilot RCT, open-label, parallel (12 wk)
Bliddal et al. 2024	Adults with obesity + knee OA (BMI ≥ 30)	Semaglutide 2.4 mg wkly + reduced-calorie diet + activity counselling	Placebo + identical lifestyle counselling	% weight change, WOMAC pain, SF-36, WC, walk distance	RCT, parallel, double-blind (68 wk + 7 wk)
Aronne et al. 2024, SURMOUNT 4	Adults with obesity (post-tirzepatide lead-in)	Tirzepatide 10/15 mg wkly + −500 kcal/d diet + activity	Placebo + identical lifestyle counselling	Weight maintenance (% loss), glycaemia, lipids, WC	RCT, withdrawal, double-blind (52 wk after 36-wk open)
Gibbons et al. 2020	Adults with T2D (BMI ~30, 87 % M)	Oral semaglutide 14 mg daily	Placebo	Ad libitum energy intake, VAS appetite, CoEQ, snack-box preference	RCT, crossover, double-blind (2 × 12 wk)
Blundell et al. 2017	Adults with obesity (BMI 30-45)	Semaglutide 1 mg wkly	Placebo	Ad libitum energy intake, VAS appetite, CoEQ, Leeds Food Preference Task, body composition	RCT, crossover, double-blind (2 × 12 wk)

Friedrichsen et al. 2021	Adults with obesity (BMI 30-45)	Semaglutide 2.4 mg wkly	Placebo	Ad libitum lunch intake, VAS appetite, CoEQ, gastric emptying, body composition	RCT, parallel, double-blind (20 wk + 7 wk)
Heise et al. 2023	Adults with obesity (post-tirzepatide lead-in)	Tirzepatide 10/15 mg wkly + −500 kcal/d diet + activity	Placebo + identical lifestyle counselling	Weight maintenance (% loss), glycaemia, lipids, WC	RCT, withdrawal, double-blind (52 wk after 36-wk open)
Johnson et al., 2025	Adults using GLP-1 RA for ≥1 month (N=69), majority female (79.7%)	GLP-1 RA therapy (Semaglutide 53.6%, Tirzepatide 33.3%, Dulaglutide 11.6%)	Dietary Reference Intakes (DRI) and MyPlate food group guidelines	Nutrient intake (macronutrients, micronutrients), MyPlate food group servings, protein intake (g/kg/day), diet quality	Cross-sectional observational study (3-day ASA24 food record)

Abbreviations: AE (Adverse Event), AIRg (Acute Insulin Response to Glucose), ASA24 (Automated Self-Administered 24-hour Dietary Recall), BMI (Body Mass Index), BP (Blood Pressure), CHO (Carbohydrate), CoEQ (Control of Eating Questionnaire), DRI (Dietary Reference Intake), F (Fat), FFM (Fat-Free Mass), FM (Fat Mass), GI (Gastrointestinal), GLP-1 RA (Glucagon-Like Peptide-1 Receptor Agonist), HbA1c (Glycated Haemoglobin), HOMA-IR (Homeostatic Model Assessment of Insulin Resistance), kcal (Kilocalorie), LCD (Low-Calorie Diet), LEKT (Low-Energy Ketogenic Therapy), LFPT (Leeds Food Preference Task), M (Male), MS (Muscle Strength), OA (Osteoarthritis), P (Protein), RCT (Randomised Controlled Trial), RMR (Resting Metabolic Rate), SF-36 (Short Form-36 Health Survey), T2D (Type 2 Diabetes), VAS (Visual Analogue Scale), VLCD (Very-Low-Calorie Diet), WC (Waist Circumference), wk (Week), WOMAC (Western Ontario and McMaster Universities Osteoarthritis Index)
Units and symbols: % (Percent), + (Plus / “with”), < (Less than), ≥ (Greater than or Equal to), → (Dose escalation), ~ (Approximately), cm (Centimetres), d (Day), g (Grams), g/kg/day (Grams per Kilogram per Day), kg (Kilogram), kg/m² (Kilograms per Square Metre), kcal/d (Kilocalories per Day), mg (Milligram), min/wk (Minutes per Week).
